# A single setup approach for the MRI‐based measurement and validation of the transfer function of elongated medical implants

**DOI:** 10.1002/mrm.28840

**Published:** 2021-05-25

**Authors:** Peter R. S. Stijnman, M. Arcan Erturk, Cornelis A. T. van den Berg, Alexander J. E. Raaijmakers

**Affiliations:** ^1^ Computational Imaging Group for MRI diagnostics and therapy Center for Image Sciences UMC Utrecht Utrecht the Netherlands; ^2^ Department of Biomedical Engineering Eindhoven University of Technology Eindhoven, Brabant the Netherlands; ^3^ Restorative Therapies Group, Implantables R&D, Medtronic PLC Minneapolis Minnesota USA

**Keywords:** measurement, simulation, transfer function, validation

## Abstract

**Purpose:**

To propose a single setup using the MRI to both measure and validate the transfer function (TF) of linear implants. Conventionally, the TF of an implant is measured in one bench setup and validated using another.

**Methods:**

It has been shown that the TF can be measured using MRI. To validate this measurement, the implant is exposed to different incident electric fields, while the temperature increase at the tip is monitored. For a good validation, the incident electric fields that the implant is exposed to should be orthogonal. We perform a simulation study on six different methods that change the incident electric field. Afterward, a TF measurement and validation study using the best method from the simulations is performed. This is done with fiberoptic temperature probes at 1.5 T for four linear implant structures using the proposed single setup.

**Results:**

The simulation study showed that positioning local transmit coils at different locations along the lead trajectory has a similar validation quality compared with changing the implant trajectory (ie, the conventional validation method). For the validation study that was performed, an *R* ^2^ ≥ 0.91 was found for the four investigated leads.

**Conclusion:**

A single setup to both measure and validate the transfer function using local transmit coils has been shown to work. The benefits of using the proposed validation method are that there is only one setup required instead of two and the implant trajectory is not varied; therefore, the relative distance between the leap tip and the temperature probe is constant.

## 1 INTRODUCTION

Strong RF fields emitted by MRI scanners may interact with active implantable medical devices (AIMDs) in patients. The AIMD is capable of enhancing the MRI RF fields, resulting in increased power deposition around the AIMD, potentially causing excessive tissue heating.^1^, ^2^, ^3^, ^4^, ^5^, ^6^ As a result, patients with an AIMD could be deprived of a valuable imaging modality.

For this reason, implant manufacturers are striving to make their products MRI safe, at least within certain predefined MRI operational constraints. Therefore, a technical specification, ISO/TS‐10974, has been developed by implant manufacturers, major MRI vendors, and the MRI community to evaluate the safety of patients with AIMDs undergoing MRI examination.^4^ Clause 8 in the technical specification describes simulation and measurement procedures that need to be performed to assess the RF heating response of AIMDs under MRI exposure conditions. One important implant characteristic described in the technical specification is the transfer function (TF).^7^ The TF decouples the scattered electric field created by the implant from the incident electric field created by the transmit RF system. When the TF for an AIMD is known, the local enhancement of the electric field can be evaluated quickly and can indicate the temperature increase that can be expected for a certain exposure condition.

The TF is especially relevant for electrically long implants (eg, AIMDs containing electrodes/lead structures) and can be obtained through either simulation or measurement. When the TF is obtained, it needs to be validated. In the conventional TF measurement setup, the electric field at the tip of the implant is monitored, while the AIMD is exposed to a local tangential electric field moving along all locations of the lead trajectory.^8^, ^9^ The electric field could be created with two sliding parallel plates, a loop coil, or an antenna, for example. During these measurements, the lead is submerged in saline water with tissue‐like conductivity.

Recently, it was shown that the TF can also be measured using an MRI system.^10^, ^11^ For this MRI‐based methodology, the concept of the transfer matrix (TM) has been introduced. The TM relates the incident electric field to the induced current along the lead and can be obtained from MRI measurements through current mapping and a model that describes the TM with only a couple of parameters. The TF is by definition the first column of the TM.

Once the TF has been obtained, a validation step is required, which can be performed through local temperature‐rise measurements. For the validation of the TF, the AIMD is typically placed in a phantom containing a high‐viscosity gel (eg, hydroxyethylcellulose gel) with the same dielectric properties in which the TF was initially measured. At the tip of the lead structure, a temperature probe is placed to monitor the temperature increase. This setup is positioned inside a realistic MRI RF transmit coil, and the implant is exposed to the incident electric field generated by this RF transmit coil. In between temperature measurements, the trajectory of the lead is altered to obtain different, preferably orthogonal, exposure conditions. Afterward, the measured temperature increase is correlated with the temperature increase calculated using the TF and the known incident electric field. This approach to validate the TF has some drawbacks.

First, to create a significantly different exposure along the lead, the lead trajectory needs to be changed in such a fashion that it may interact with itself. Therefore, the electric field created by the lead at one position can couple into the lead at another position. This effectively alters the TF of the lead, which is unwanted, as it is the quantity that needs to be validated.^12^ Simply shifting the straight lead to a different location in the phantom is not sufficient to create a different exposure condition.

The second problem is the placement of the temperature probe at the tip of the lead. For the measurements to be effective, the relative distance between the temperature probe and the lead tip needs to be consistent and preferably within a few millimeters of each other. Keeping the relative distance constant is important because the temperature hotspot that is created is highly localized and the temperature gradient is steep.^13^

Therefore, an alternative method has been presented, in which instead of changing the trajectory of the lead within the phantom, a multitransmit coil array is used to alter the incident electric‐field distribution using a large set of phase‐amplitude drive settings.^12^ This method could also be achieved with a dual‐transmit birdcage body coil. The downside of using this method, however, is that the incident electric fields are often very similar. Such a setup can only generate a fixed potential maximum number of exposure conditions that are equal to the number of elements in the array. Of course, these systems have the ability to impose an infinite variation of field distributions on the implant using varying phase and amplitude settings. However, these field distributions are superpositions of the fields created by the independent elements of the array and are therefore linearly dependent.

There are also other options to create different exposures along the lead trajectory for the validation of the TF instead of altering the trajectory itself. Here, we will be aiming at validation methodologies that use an MRI setup that would enable the MRI‐based measurement and subsequent validation in one session. The first alternative method would be to move the phantom with respect to the birdcage coil, and with it, its associated electric‐field distribution.

The second option is to drive a dual‐transmit birdcage body coil in various phase‐amplitude combinations.^14^ However, as discussed, this will only provide a maximum of two orthogonal exposure conditions. This method could still be combined with one of the other methods described here.

The third option is to use passive scatterers to change the exposure. A first example is dielectric padding.^15^, ^16^ These dielectric pads have a high permittivity and are placed between the coil and the patient or phantom and change the incident RF fields. Similar effects may be observed for materials with high conductivity and resonant structures (eg, a tuned loop). These passive scatterers could be placed at different positions along the lead to locally vary the incident electric field.

The final option, similar to the multitransmit coil array, would be to use small local transmit RF coils to apply a localized exposure that could also be placed at different positions along the lead trajectory without moving the lead itself^17^, ^18^, ^19^, ^20^ (ie, moving the transmit coils rather than changing the lead trajectory), which facilitates a constant relative distance between the lead tip and the temperature probe. This entails that the birdcage transmit coil be used for the TF measurement, and the local transmit coils would be used for the validation of that TF measurement.

In this study, we aim to develop a new validation procedure that is particularly suitable for validating MRI‐measured TFs. The study consists of two parts: First, we simulate the methods described previously to identify the best validation methodology. Second, we use the best method to perform a complete experimental workflow of MRI‐based TF measurement and validation for four elongated structures: two copper wires (ie, one bare and one insulated), a realistic coaxial implant lead, and a spinal cord stimulator.

## 2 METHODS

### 2.1 Simulation‐based investigation of potential validation methods

In section 2 we will describe how the selected validation methods have been simulated, and we define a metric to assess the different validation methods called the validation quality.

All methods are evaluated for the validation quality of a nonspecified structure of 40‐cm length that is placed within an ASTM phantom. The phantom is filled with hydroxyethylcellulose gel with a relative permittivity of 78 and a conductivity of 0.34 S/m.

**FIGURE 1:**
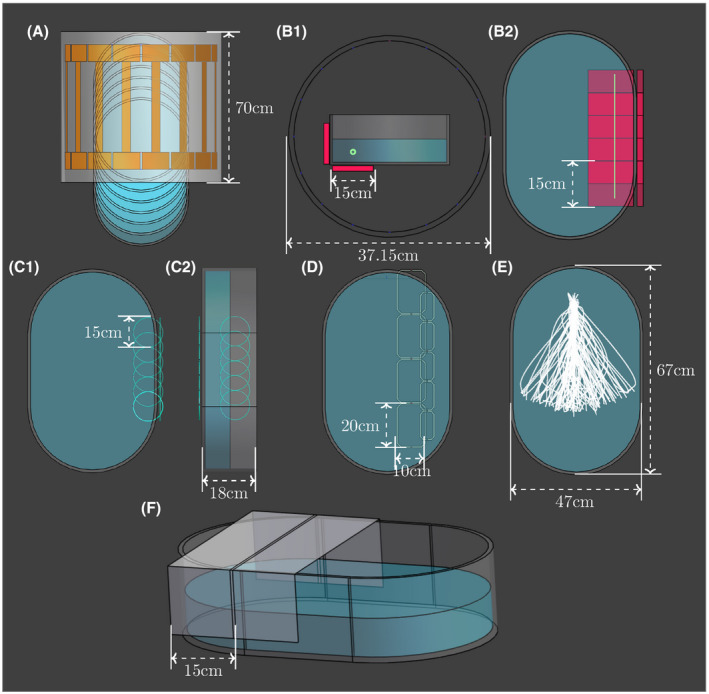
All of the simulation setups that were used to investigate the methodologies to change the incident tangential electric field. A, The phantom was shifted out of the birdcage coil. B1,B2, The side and top view of the dielectric pads. The electric field is extracted at the location of the light green trajectory; this location within the phantom is used for all methodologies except changing the trajectory. C1,C2, Top and side view of the passive RF coil positions. D, The local transmit coil positions. E, The 100 random implant trajectories that were extracted. F, Example of how the phantom was wrapped in aluminum foil

The intended validation method setup is simulated using the numerical electromagnetic simulation package *Sim4Life* (v5.0; ZMT, Zurich, Switzerland). All methods except the local transmit method make use of the birdcage body coil of the MRI system. The dimensions of the birdcage coil, the ASTM phantom, and all other parts used for the validation methods are indicated in Figure 1.

#### 2.1.1 Shifting the phantom

As mentioned in section 1, it is possible to create different exposure conditions using a dual‐transmit birdcage coil and shifting the phantom along the bore axis with respect to the coil. It begins with the ASTM phantom centered inside a birdcage coil and shifting it 50 mm outward of the coil along the center bore axis to a maximum of 300 mm.

#### 2.1.2 Passive scatterers

This category consists of three different methods: dielectric pads, aluminum foil shielding, and passive resonant RF loops. A dielectric pad with a relative permittivity of 450 is used to create different exposure conditions. The placement of the aluminum sheets is shown in Figure 1 and Supporting Information Figure S1. A total of 10 configurations were simulated. The resonant loop coil has a thickness of 3 mm, a capacitor value of 28.6 pF, and placed at the same locations as the dielectric pad. For all three methods described previously, the birdcage coil was simulated as a multiport simulation to obtain twice the number of exposures.

#### 2.1.3 Local transmit coils

We simulated two rectangular local transmit loop coils made with strips, simulated as a perfect electrical conductor. The loop coils are tuned to 64 MHz using two capacitors and are placed along different positions of the lead trajectory.

### 2.2 Validation quality

To adequately validate the measured TF, the incident tangential electric fields that the lead is exposed to during the validation should be linearly independent with respect to each other. A method in which a higher number of linearly independent exposure conditions can be realized means it is more suitable. For example, electric‐field distributions that always have the same amplitude and phase distribution, and are only scaled globally, are not linearly independent and would result in a poor validation quality. A better set of distributions preferably has many linearly independent incident electric‐field distributions.

To assess which validation method performs best in terms of the validation quality and effectiveness, we use the singular value decomposition (SVD). The SVD will decompose any matrix into orthogonal vectors and a matrix containing singular values. Consider a matrix that consists of a collection of vectors as its columns. The singular values associated with this matrix indicate the lengths of the orthogonal vectors. If many of the original vectors are linearly dependent, the singular values will quickly decrease to zero. This entails that the original matrix be described predominantly by a small number of orthogonal vectors. If the vectors are linearly independent, the singular values will be higher and the original matrix can only be adequately described using a larger number of orthogonal vectors.

For the validation methods that we simulated, the set of incident tangential electric‐field distributions is extracted along the implant trajectory (when the implant is not present). These different exposures are first normalized to equal vector length and then all placed as columns into a matrix. This matrix is then decomposed using the SVD in which the resulting singular values are normalized and summed to obtain a single metric for the validation quality, where a higher number indicates a better validation (ie, more linearly independent exposure conditions).

### 2.3 Proof‐of‐principle validation study using the best performing MRI‐setup validation method

**FIGURE 2:**
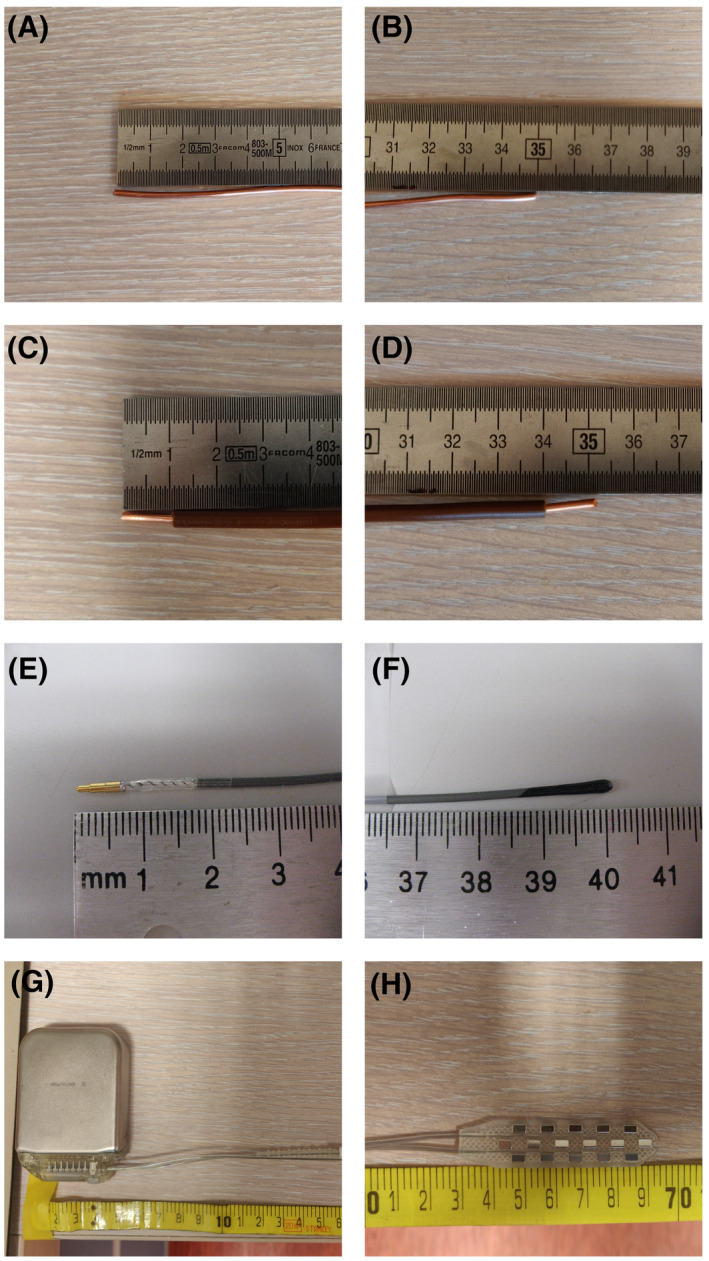
The ends of the implants for which the transfer functions (TFs) were measured and validated. A,B, Both ends of the bare copper wire. C,D, Both ends of the insulated copper wire where the ends of the insulation are removed for about 1 cm. E,F, The ends of the coaxial lead and a proper lead tip. G,H, The Implantable Pulse Generator and the electrode patch of the spinal cord stimulator, respectively

The second part of this study will focus on performing a combined TF measurement and validation study for four linear implants. The first is a 36‐cm bare copper wire; the second is a 36‐cm insulated copper wire; and the third is a 40‐cm coaxial implant lead (that was supplied to us by Medtronic [Fridley, MN]) with a tip structure on one end, while the other end is insulated. The last implant is a spinal cord stimulator (Prime Advanced Sure Scan MRI; Medtronic) with an Implantable Pulse Generator at one end and an electrode array at the other end. Furthermore, there are two leads of 70 cm running from the Implantable Pulse Generator to the electrodes. The diameter of the copper wires is 2.5 mm, the insulation layer has a thickness of 1 mm, and the diameter of the coaxial lead is 1.25 mm. The lead structures are shown in Figure 2.

The phantom that was used in this measurement and validation study is the same ASTM phantom that was used in the simulation‐based study described in section 1.

The leads and the temperature probe are suspended inside the phantom using plastic screws and nylon threads. Fixating the leads and the temperature probe enables them to be close to each other and not drift away or sink to the bottom of the phantom, all the while maintaining the signal around the lead intact for MRI measurements. Particular care has been given to ensure that the temperature probe cannot move relative to the lead tip.

### 2.4 Magnetic resonance imaging–based transfer function measurement

For the transfer function measurement, the procedure has been followed as described by Tokaya et al.^11^ This procedure makes use of the TM of the implant. To obtain the TM from an MRI experiment, the induced current (*I^ind^*) and the incident tangential electric field ($E_{\tan}^{i}$) are required to minimize

(1) $$f={∥I^{\mathrm{ind}}‐TME_{\tan}^{i}∥}_{2}^{2},$$

where TM is the transfer matrix. Using an attenuated wave model, the matrix is parametrized by only a few unknowns (ie, 6 to 10 for the lead structures defined here).^11^ The magnitude of the induced current and the phase difference between the current and the background $B_{1}^{+}$ are calculated from the measured $B_{1}^{+}$ magnitude distribution around the lead. This $\left|B_{1}^{+}\right|$ distribution is obtained using a fast field‐echo (FFE) series with variable flip angles. For this work, we used finite‐difference time‐domain simulations to obtain the incident tangential electric field and the phase information of the incident $B_{1}^{+}$ (ie, this phase information together with the measured phase difference is used to calculate the phase of the induced current) field to fit the TM.^11^

### 2.5 Transfer function simulation

For reference, the TF of the four investigated leads was also simulated using the same EM modeling package *Sim4Life*. We obtained simulated TFs by performing the MRI‐based TF measurement in silico, as outlined by Tokaya et al.^11^ These TFs were obtained from simulated $B_{1}^{+}$ fields rather than measured $B_{1}^{+}$ fields. The same geometries as in the actual MRI‐based measurements were used.

### 2.6 Validation method

To validate the TF using temperature measurements, we correlate the SAR from the temperature measurement and the SAR we calculate using the TF and the incident tangential electric field obtained from simulations. To calculate the actual SAR from the temperature measurement, we use the fact that the initial slope of the temperature increase is proportional to the SAR, as follows^21^:

(2) $$SAR=c\frac{\partial T}{\partial t}{|}_{t=0},$$

where *c* is the specific heat capacity of the tissue surrounding the lead tip; *T* is the temperature; and *t* is the time. The temperature was sampled every 0.7 seconds. To accurately obtain the initial slope of the temperature increase, we fitted a set of exponential growth and decay functions to the temperature curve.^22^ This fit allows us to use all of the data points of the curve.

To calculate the specific absorption rate (SAR) using the TF, we first calculate what the scattered electric field at the tip of the lead is, given the incident tangential electric field. Then, together with the incident electric field created at the tip by the source, we can compute the SAR at the lead tip by

(3) $$SAR\propto \sigma {\left(\alpha_{1}\left|E^{\mathrm{sc}}\left|+\right|E^{i}\right|\right)}^{2},$$

where *σ* is the conductivity of the surrounding dielectric, and *α*_1_ is the calibration coefficient we need to compute, as the TFs that are measured are all normalized. This calibration coefficient is calculated using linear regression between the predicted SAR using the TF and the measured SAR from the temperature probes. Afterward, we can compute the *R* ^2^ for this linear regression and find out how well the TF describes how the implant reacts to the incident fields.

### 2.7 Validation measurement

For the validation of the TF, we used local transmit coils to create the incident RF fields and varied the incident electric field by shifting the transmit coils along the lead between different heating tests. The transmit coils that were constructed had the same dimensions as the ones simulated. The transmit coils were tuned to 64 MHz and matched using a vector network analyzer. The measurement series was performed once with the smaller coil and once with the larger coil. For the larger coil, it was moved in steps of 6 cm, and the small coil in steps of 4 cm. This in total created 10 different measurements per lead structure. To connect the local transmit coils to the MRI scanner, the birdcage coil was unplugged from the quadrature hybrid power unit and connected with dummy loads. Afterward, the local transmit coil was connected to one of the quadrature hybrid output channels, while the other channel was connected to a dummy load.

The temperature increase is measured using fiber optic temperature probes (OpSens, type OTP‐M; resolution, 0.01k; accuracy, 0.30k [99.9% confidence level]) that can be used inside the MRI system (Ingenia; Philips, Best, the Netherlands). To accurately measure the temperature increase as a result of the lead only, a temperature probe at the tip of the lead and a reference background temperature probe is required. The temperature probe at the tip of the lead will measure the temperature increase as a result of the incident and scattered electric, while the reference temperature probe is positioned away from the lead. This reference temperature probe will measure the temperature increase that is associated with only the incident electric field. The temperature probe at the tip of the lead is placed close (less than 2 mm away) to the tip of the lead, to obtain a reliable correlation.^13^

To generate sufficient heating for the probes to register and for us to predict, we use the MRI system to deposit power into the phantom. This was achieved with a turbo spin echo with a long echo train (ie, 25 180° RF pulses). The RF exposure was continued until significant heating occurred and enough time passed to obtain a good fit (preferably 0.5 K to 1.5 K) or the temperature increase started to flatten.

**FIGURE 3:**
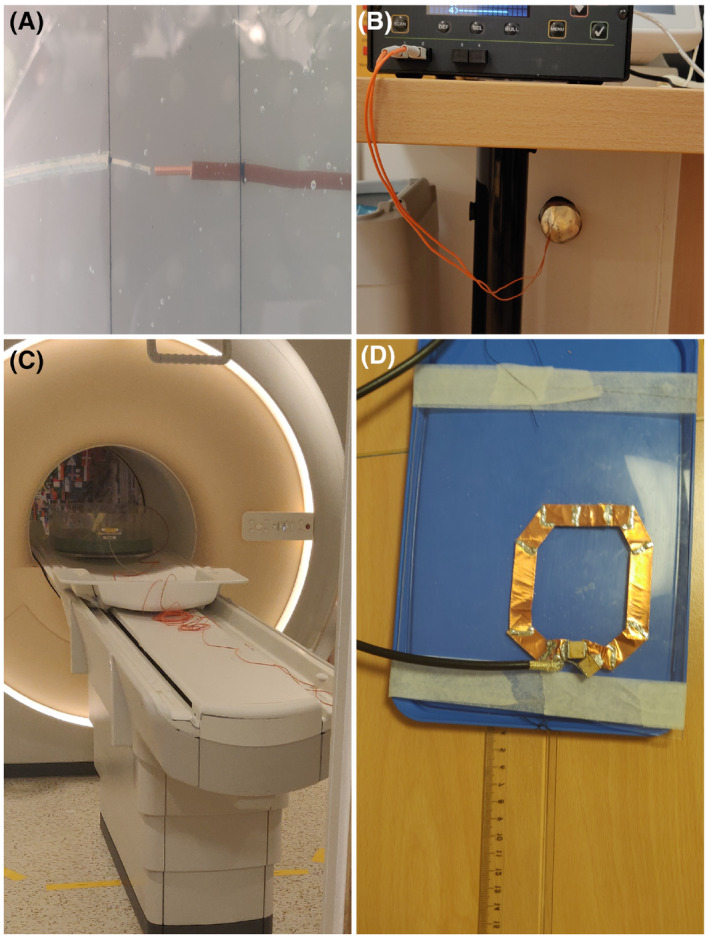
The measurement setup. A, The positioning of the temperature probe with respect to the lead tip. B, The temperature measurement device. C, How the probes are entered through the RF waveguide into the MRI room. D, Using the ruler, we measured the distance from the end of the ASTM phantom toward the place of the local transmit coil when it is placed underneath the phantom

Figure 3 shows the temperature measurement setup. This includes the position of the temperature probe relative to the lead tip, the temperature measurement equipment, and how the phantom is placed inside the MRI. The last picture in the figure shows how the placement of the coils was measured with respect to the end of the ASTM phantom.

### 2.8 Uncertainty analysis

From the MR and temperature measurements, we obtain a single predicted and measured SAR value. However, both measurements are subject to noise that corrupts the underlying data, which affects the resulting SAR values that are found. Therefore, we performed an uncertainty analysis for both the MR and temperature measurements. The result was a SD around the obtained SAR values that showed the accuracy of the proposed methodology.

**FIGURE 4:**
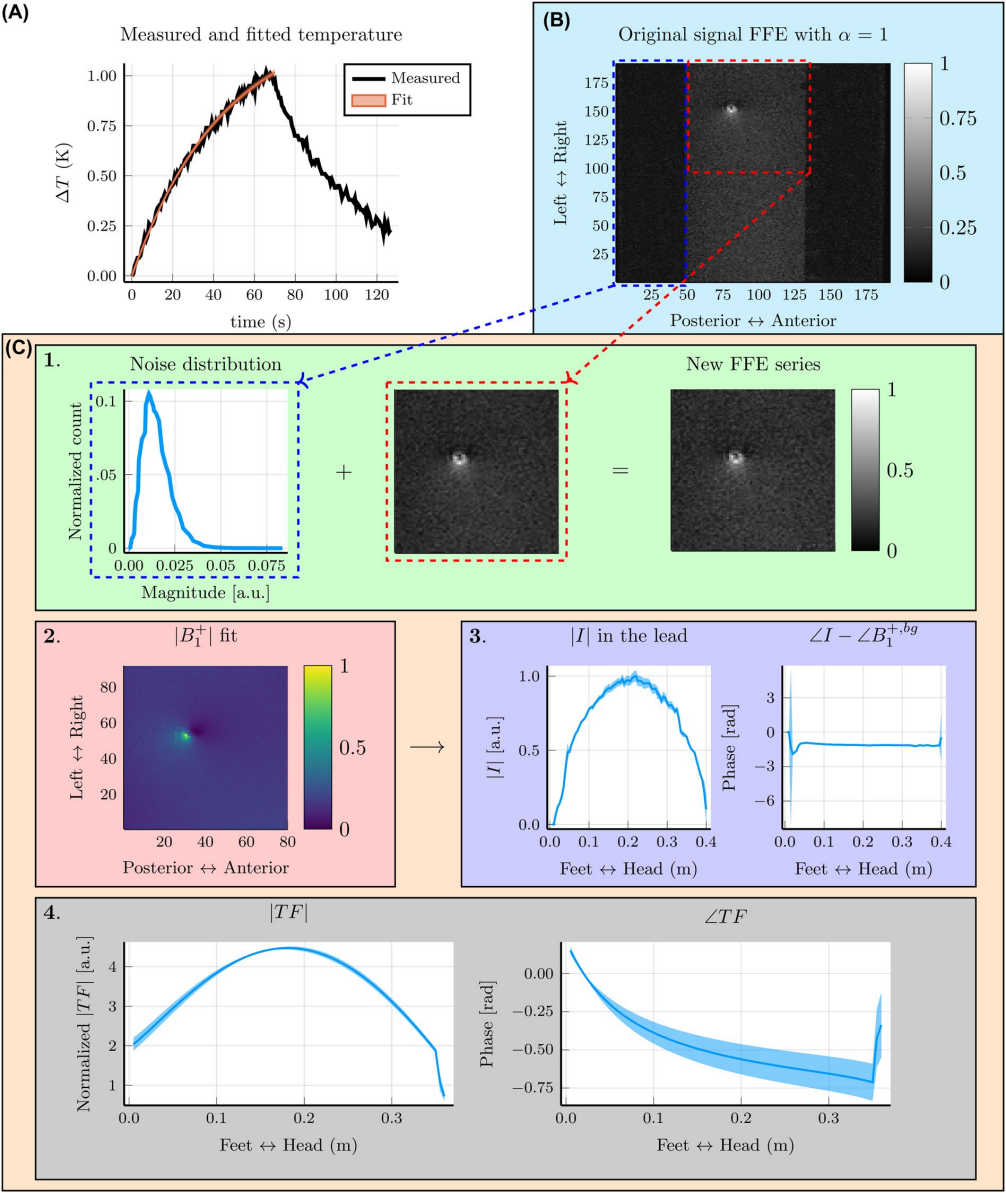
Overview of the uncertainty analysis. A, The curve fit with uncertainty of a single temperature measurement; this fit is used to find the measured specific absorption rate (SAR). B, Transverse slice of the fast field‐echo (FFE) sequence, where the artifact created by the lead is visible. C, The steps in the Monte Carlo simulation approach find the uncertainty of the measured TF, and thereby the uncertainty in the predicted SAR. Step 1 is used to sample the noise distribution and that noise to the original FFE series to create a new FFE series. Step 2 is used to fit the $\left|B_{1}^{+}\right|$. Step 3 is used to fit the current from the $\left|B_{1}^{+}\right|$. In step 4 we fit the TF using the current and the simulated *E_tan_*. The blue‐shaded area shows the SD of the measured TF

For the temperature measurements, we fit an exponential curve using two parameters. From the fit, we calculated the Jacobian and used that to find the covariance matrix. The diagonal of the covariance matrix is the squared SDs of the fitted parameters. These SDs are used to calculate the uncertainty of the measured SAR values. This can be interpreted as a range of exponential curves that go through the measured temperature data (Figure 4A).

The predicted SAR uncertainty is determined through a Monte Carlo simulation. We first obtained the noise distribution from the FFE series measured with the MR system. From that noise distribution, we sampled new noisy data and added that to the original FFE series (Figure 4B) to create a new FFE series. Next, we went through the same step as before to obtain the TF. This included first a $\left|B_{1}^{+}\right|$ fit, then a current fit, and finally a TF fit. These steps are visualized in Figure 4C. For all of the leads shown in this paper, this process was done 100 times. From the found TFs and the simulated electric field, we obtained a range of predicted SAR values.

## 3 RESULTS

### 3.1 Simulation‐based investigation of potential validation methods

**FIGURE 5:**
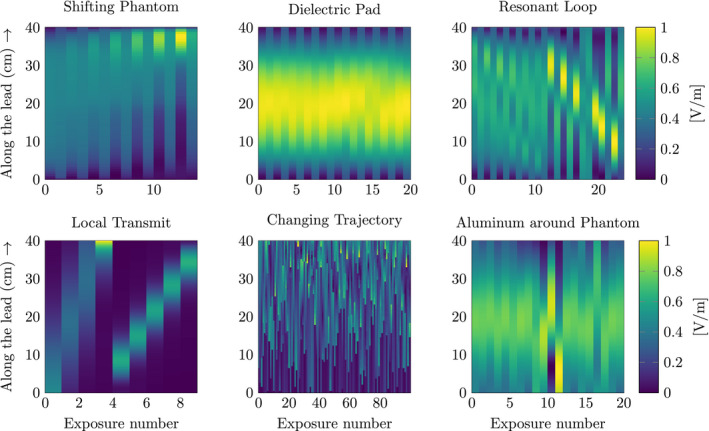
The incident tangential electric fields for all of the described methods, to alter the exposure conditions. Each column represents the electric field along the implant trajectory. The columns are normalized to have the same vector length. Afterward, these matrices are used to compare the normalized singular values between the methods

Figure 5 shows the magnitudes of the matrices on which the SVD is applied. Each column of the matrices corresponds to the magnitude of the electric field along the implant location, which is extracted from the finite‐difference time‐domain simulations. One column in the matrix corresponds to one simulation. It can be observed that shifting the phantom, wrapping the phantom in aluminum, and using dielectric pads do not alter the electric field along the lead significantly. Adding passive resonant loop coils shows more spatially varying exposures. For the local transmit coils and bending the lead, we observe the largest changes in the incident electric‐field distribution along the leads.

**FIGURE 6:**
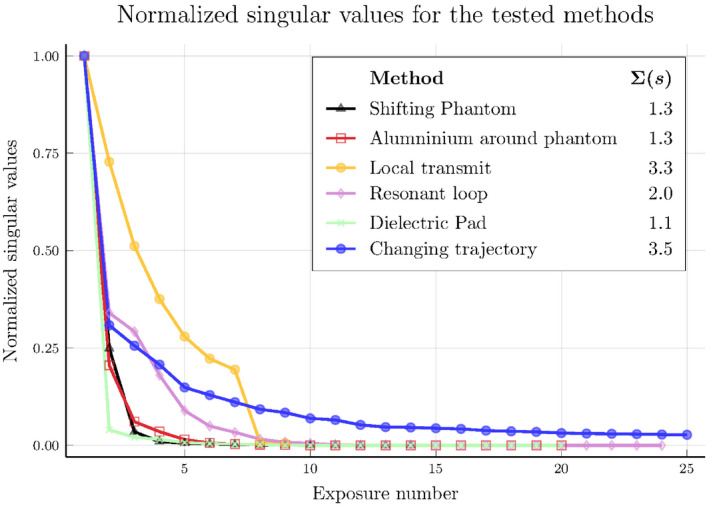
The normalized singular values of the different excitation methods. The legend indicates the sum for the different methods, where a higher number indicates more equivalent orthogonal exposure conditions; thus, more information is obtained using that method

After the SVD has been applied to the matrices in Figure 5, the singular values are extracted and compared with each other in Figure 6. Similar to the observations mentioned previously, we find that the singular values for the methods (shifting the phantom, wrapping the phantom in aluminum, and using dielectric pads) decay rapidly. For the passive resonant loop coil and changing the trajectory, this decay of the singular values is slower. Finally, the singular values for the local transmit loop decay the slowest. The slower the decay of the singular values, the more suitable the validation method is for generating orthogonal exposure conditions.

The validation quality of the presented methods is expressed in the sum of these normalized singular values and can be observed in Figure 6. This sum indicates how many equivalent orthogonal exposure conditions are subjected to the lead, where a higher sum indicates that more information is obtained from conducting those experiments. Figure 6 also shows that the sum of the normalized singular values for shifting the phantom, wrapping the phantom in aluminum, and using dielectric pads is small (ie, 1.3, 1.3, and 1.1, respectively). For the resonant loop, the validation quality increases to 2.0, but it is still low compared with using local transmit coils at different positions (3.3) and changing the trajectory (3.5). It could be concluded that changing the trajectory will result in the best validation of the TF; however, more temperature measurements are required. Furthermore, local transmit coils have the added benefit that the distance between the temperature probe and the lead tip is constant.

### 3.2 Proof‐of‐principle validation study using best performing MRI‐setup validation method

**FIGURE 7:**
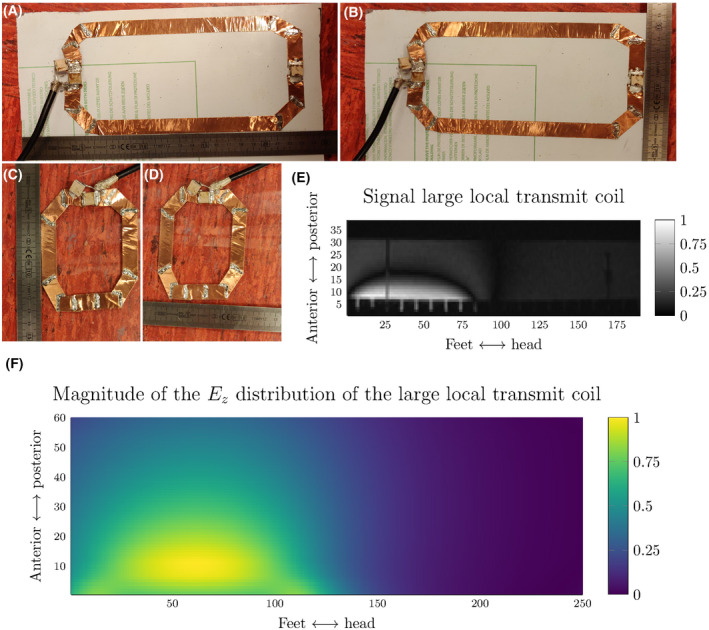
The two local transmit coils that were constructed to create different incident tangential electric field exposures along the lead trajectory. A,B, The length and width of the larger loop coil. C,D, The length and width of the smaller loop coil. E, The generated signal of the larger loop coil in a sagittal slice through the phantom. F, The corresponding z‐component of the electric field

Because of the higher validation quality and the fact that we want to keep the relative distance between the lead tip and the temperature probe the same, the choice was made to construct the local transmit loop coils and use them to create different exposure conditions in the heating test to validate the TFs. These constructed loop coils are depicted in Figure 7. The smaller coil is on a transparent plastic substrate.

**FIGURE 8:**
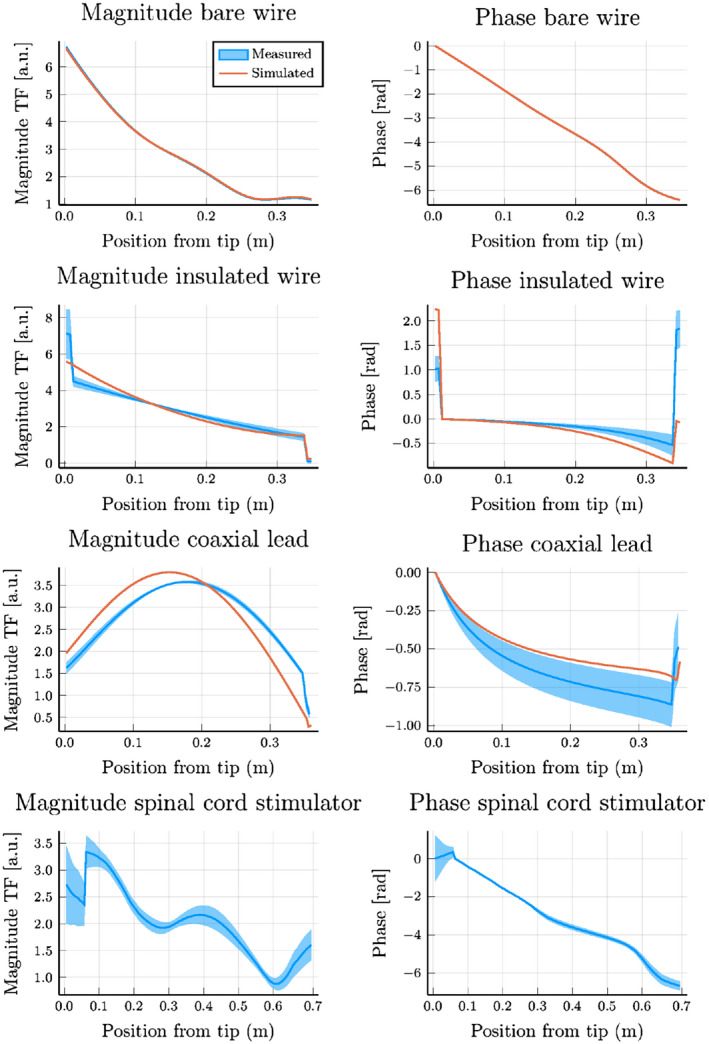
A comparison between the transfer functions obtained with finite‐difference time‐domain simulations and the transfer function obtained with MRI. The top row shows the magnitude and phase of the transfer function for the bare copper wire; the second row shows the same for the insulated copper wire; the third row shows the TF for the coaxial lead; and the bottom row is the TF for the spinal cord stimulator. For all of the TFs, the blue‐shaded area displays the SD of the measured TF

First, the TF was measured for the three investigated structures (Figure 2) using a 1.5T MRI system. The resulting TFs are shown in Figure 8 along with their simulated counterparts for reference.

Using the setup depicted in Figure 3, we obtained the temperature increases at the tip of the lead, as shown in Figure 4A. The temperature measurement is indicated in black and the fitted temperature in orange. Between measurements, the position of the local transmit coils was shifted to a different position along the lead trajectory.

**FIGURE 9:**
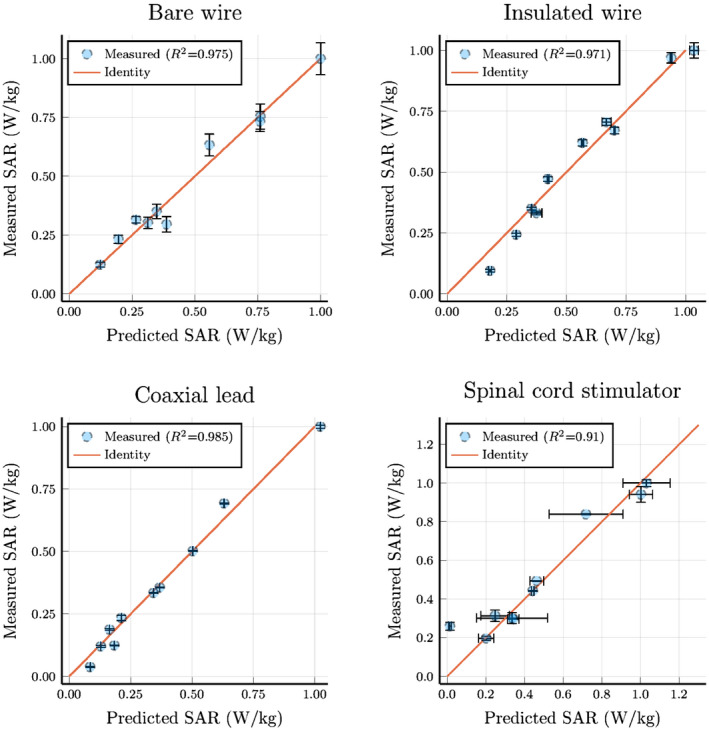
The SAR that is calculated using the transfer function and the known exposure condition correlated with the SAR that is calculated from the measured temperature curves. Six measurements were done with the small transmit coil, and four were done with the large transmit coil for a total of 10 measurements per lead

Finally, the SAR is obtained from the temperature measurement by fitting an exponential to accurately determine the derivative at the start of the RF exposures. This will be referred to as the measured SAR. Furthermore, we can calculate the SAR using the measured TFs and the simulated incident tangential electric fields, which we refer to as the predicted SAR. The SAR values were normalized to 1 W/kg for all of the tested leads, as the exposure conditions we subjected the leads to are not to be expected during a regular MR examination. These results are plotted in Figure 9, where we used linear regression to find the calibration coefficient (*α*_1_) that maximizes *R* ^2^, which resulted to be equal to 0.91 at a minimum.

## 4 DISCUSSION

Previous work has demonstrated that the TF for linear implants can be acquired with the use of MRI through the image artifact created in the RF transmit fields by the implant. However, in a typical RF safety assessment procedure, the measured TF needs to be validated too. Conventionally, the validation of the TF is performed by changing the implant trajectory within a phantom inside a birdcage body coil and measuring the temperature increase at the tip of the implant lead. This methodology has some disadvantages and is difficult to implement using the same setup as the MRI‐based TF measurement. Therefore, we aim to define a validation procedure that can be incorporated into the same setup as for the MRI‐based measurement of the TF, which would expedite the entire process.

The first part of the paper consists of a simulation‐based study in which we compare different TF validation methods. To compare the different methodologies in terms of their validation effectiveness, we used the SVD. The SVD decomposes the matrix of incident field distributions of the evaluated methods into a set of orthogonal vectors with corresponding singular values. Normalizing and summing the singular values results in the number of equivalent orthogonal incident field distributions that the method is capable of creating. The more orthogonal incident field distributions that can be created, the higher the validation quality of that method.

From the simulation study, we found that shifting the phantom inside the MRI system using dielectric pads, and wrapping the phantom in aluminum foil, are not effective in creating orthogonal incident field distributions. Using a passive RF coil is more effective, but still falls short with respect to the conventional validation method. Positioning local transmit coils at different positions along the implant trajectory has comparable effectiveness compared with changing the implant trajectory. This method has the added experimental benefit that the temperature probes stay at the same position relative to the lead tip.

Figure 6 shows that the singular values for the local transmit coils drop rapidly after the seventh exposure. Therefore, increasing the number of positions along the implant trajectory will have little effect in terms of validation quality. To increase the validation quality further, a third transmit coil could be constructed that creates a spatially different electric field. A benefit, however, is that the number of temperature measurements that are required for the proposed validation is significantly fewer compared with the conventional method (ie, 9 compared with 100 temperature measurements).

For the second part of the paper, we completed an MRI‐based TF measurement and validation procedure for four elongated lead structures using the validation method that emerged as the best candidate from the simulation comparison: local transmit coils.

The validation procedure resulted in the scatter plot depicted in Figure 9, which shows a good correlation ($R^{2}\geq 0.91$) between the measured and predicted SAR at the tip of the lead trajectory. This means that for a given incident electric field (ie, in this case, created by local transmit coils) and the MRI‐measured TF, the predicted SAR is in agreement with the independently measured temperature increase. The reason we find these high coefficients of determination is a result of multiple beneficial factors compared with the conventional methodology.

The first benefit is that the used setup for the measurement of the TF is the same as the setup for the validation. The phantom, the implant position within the phantom, and its orientation are all exactly the same. Furthermore, because the implant trajectory is kept constant and straight, the implant cannot couple with itself and effectively alter the TF.

Another important experimental benefit for the presented validation method is that the relative distance between the lead tip and the temperature probe is constant, as we move neither. This results in all of the temperature measurements being in the same position, eliminating any errors as a result of the misplacement of the temperature probe. These errors can be quite large, as the gradient of the temperature elevation around the tip is very steep.^13^, ^23^ The final major benefit of using this measurement and validation method is that only one setup is required to be built. This decreases the effort significantly and reduces the possible number of errors that can be made.

A potential caveat to this method is that there might be coupling between the implant and the local transmit coil. This could alter the TF and induce errors during the validation. Therefore, this potential error source was investigated using finite‐difference time‐domain simulations. We simulated the TF for the insulated copper wire with and without the local transmit coil placed next to the phantom. Negligible changes were observed between the two TFs (Supporting Information Figure S2).

The presented validation method also contains some drawbacks. The major drawback is that the local transmit coils that are used for the validation have to be simulated precisely to obtain the correct incident tangential electric field. Otherwise, the wrong SAR is predicted and the measured and predicted SAR might not correlate anymore. Thus, any errors made in the simulation of the local transmit coils will be propagated to errors in the validation of the TF. For the loop coils, the RF fields are smooth and predominantly affected by the dimensions of the coil itself. Simulating other types of coils or antennas might be more difficult and prone to errors.

Another drawback of this validation methodology is that the coils are connected to the quadrature hybrid of the birdcage body coil. Although the procedure is not difficult, it can be a considerable obstacle for some institutes or systems. More ideal would be to have a local transmit coil with more appropriate coil interfacing. Although the method has been designed specifically to facilitate the validation of MR‐based measured TFs, the validation method could also be applied outside of the MR scanner with a separate power source for the coils. Combined with a benchtop TF measurement setup, this could be a cheaper solution than using the MR system. However, depending on the setup, the phantom may need to be moved between the TF measurement and validation, which could possibly displace the lead or the temperature probe.

The uncertainty depicted in Figure 9 shows that, overall, the presented method is accurately predicting the SAR at the tip of the leads. Only for the spinal cord stimulator, we see larger uncertainties arising for the measured TF. This can be a result of the more complicated structure of the implant. Another reason for the larger uncertainty is that the amount of usable data in the FFE sequence was limited, because parts of the signal were corrupted by small ferromagnetic parts inside the implant. The uncertainty in the measured SAR is significantly increased when the scans used for heating were terminated prematurely or when the overall temperature increase was small.

The uncertainty analysis includes the goodness of fit for the temperature data for the measured SAR and the data uncertainty for the TF fit. The simulated electric fields, the positioning of the local transmit coils, and any model imperfections are not included. These uncertainties have been investigated in detail by Neufeld et al, who found the uncertainty in the SAR arising from differences in conductivity, permittivity, phantom placement, and implant placement to be in the order of 5.6%,^24^ where changes in the conductivity and permittivity varied a percent and contributed to the largest source of uncertainty. The phantom placement was varied 10 mm in all three directions, and the implant placement varied 1 mm in all directions. The implant placement could be equated to our local transmit coil placement, as the positions are relative to each other. Therefore, the actual uncertainty depicted in Figure 9 is likely higher; however, these values give an impression of the accuracy of the presented methodology.

In this work, we only validated the TF of the tip of the four leads. The TFs of other electrodes in the patch of the spinal cord stimulator can be validated using this methodology. This can be done using the column of the TM that corresponds to the electrode location and placing a fiberoptic temperature probe at that electrode. For the TF for the RF rectification voltage at the Implantable Pulse Generator header of the spinal cord stimulator, the last column of the TM should be used; however, this TF cannot be validated using this method, because the temperature probes cannot be placed inside the Implantable Pulse Generator.

## 5 CONCLUSIONS

First, we performed a simulation study on the effectiveness of different methods to validate the TF for linear implants. We introduced a metric to assess the validation quality/effectiveness using the SVD. From the simulation study, we found that positioning local transmit coils at different locations along the fixed implant trajectory has a similar validation quality compared with the conventional method (ie, changing the implant trajectory).

Next, we performed a TF measurement and validation study on four linear implants. Here the TF is both measured and validated using the MRI system, all in one setup. The validation was done with fiberoptic temperature probe measurements, in which the incident field distribution was varied by placing the local transmit coil at different positions between heating tests. We obtained a good agreement between the predicted SAR using the TF and the measured SAR, extracted from the temperature measurements. For the investigated lead structures, using linear regression, we found an *R* ^2^ of at least 0.91. The benefits of using the presented measurement and validation method are that only one setup is required, the measured TF is not altered during the validation through self‐coupling of the lead, and the relative distance between the temperature probe and the lead tip is constant, as neither is moved during validation.

## CONFLICT OF INTEREST

M. Arcan Ertutk is a full‐time employee of Medtronic.

## Supporting information


**FIGURE S1** The different setups of the aluminum foil shielding. A, The case without any shielding. B‐D, Increasingly more aluminium foil shielding, where the foil is placed once on top of the phantom and once on the bottom of the phantom. E, Back of the phantom covered in aluminum foil; top view and side view for simulation setup. F,G, Top and side views for increasingly more aluminum foil
**FIGURE S2** A, Top view of the simulation setup. B, Side view, where the local transmit coil is placed directly underneath the phantom. The transfer function (TF) was simulated for the setup shown in (A) and (B), with and without the local transmit coil present. Below the setup, the magnitude and th phase of the simulated TFs are shown, which are in good agreement (only a negligible change in the phase)
