# Supplementary material for: A single setup approach for the MRI‐based measurement and validation of the transfer function of elongated medical implants
Source: Magn Reson Med. 2021 May 25;86(5):2751–65. doi: 10.1002/mrm.28840 (PMC8596675; doi:10.1002/mrm.28840)
Supplement: Supplementary file 1 — FIGURE S1 The different setups of the aluminum foil shielding. A, The case without any shielding. B‐D, Increasingly more aluminium foil shielding, where the foil is placed once on top of the phantom and once on the bottom of the phantom. E, Back of the phantom covered in aluminum foil; top view and side view for simulation setup. F,G, Top and side views for increasingly more aluminum foil FIGURE S2 A, Top view of the simulation setup. B, Side view, where the local transmit coil is placed directly underneath the phantom. The transfer function (TF) was simulated for the setup shown in (A) and (B), with and without the local transmit coil present. Below the setup, the magnitude and th phase of the simulated TFs are shown, which are in good agreement (only a negligible change in the phase) [file MRM-86-2751-s001.pdf]

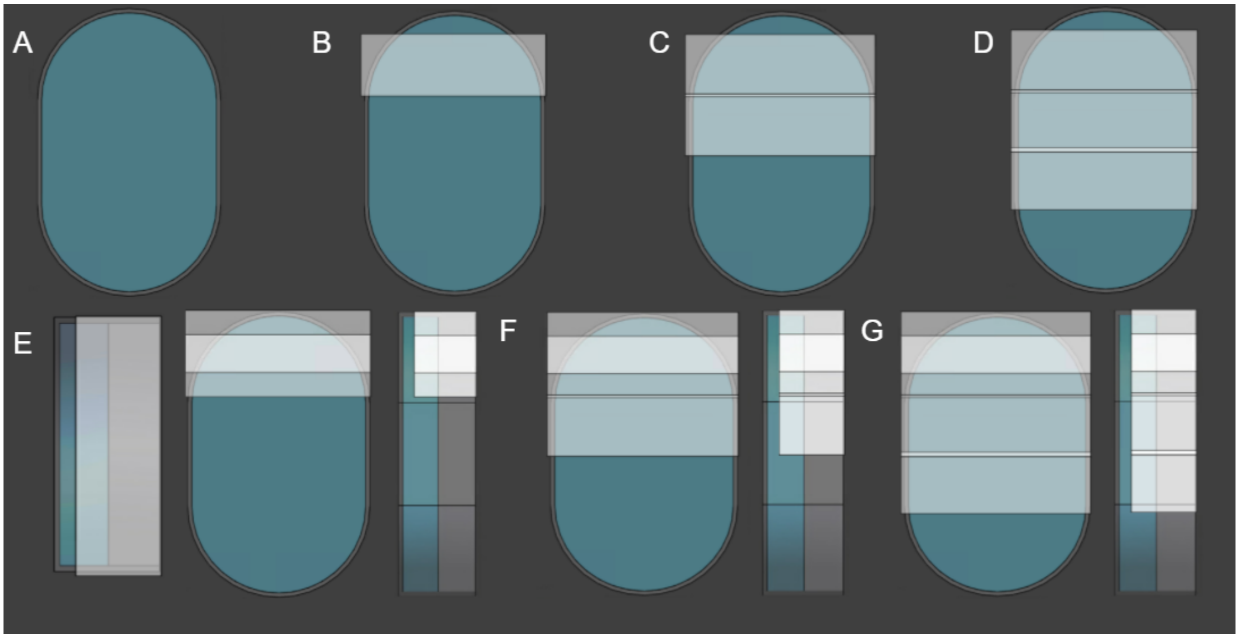

Supporting Information Figure S1: The different setups of the aluminium foil shielding. A) shows the case without any shielding. B) & C) & D) show increasingly more aluminium foil shielding where the foil is placed once on top of the phantom and once on the bottom of the phantom. E) shows first the back of the phantom covered in aluminium foil then the top view and the side view for once simulation setup. F) & G) show the top and side view for increasingly more aluminium foil.

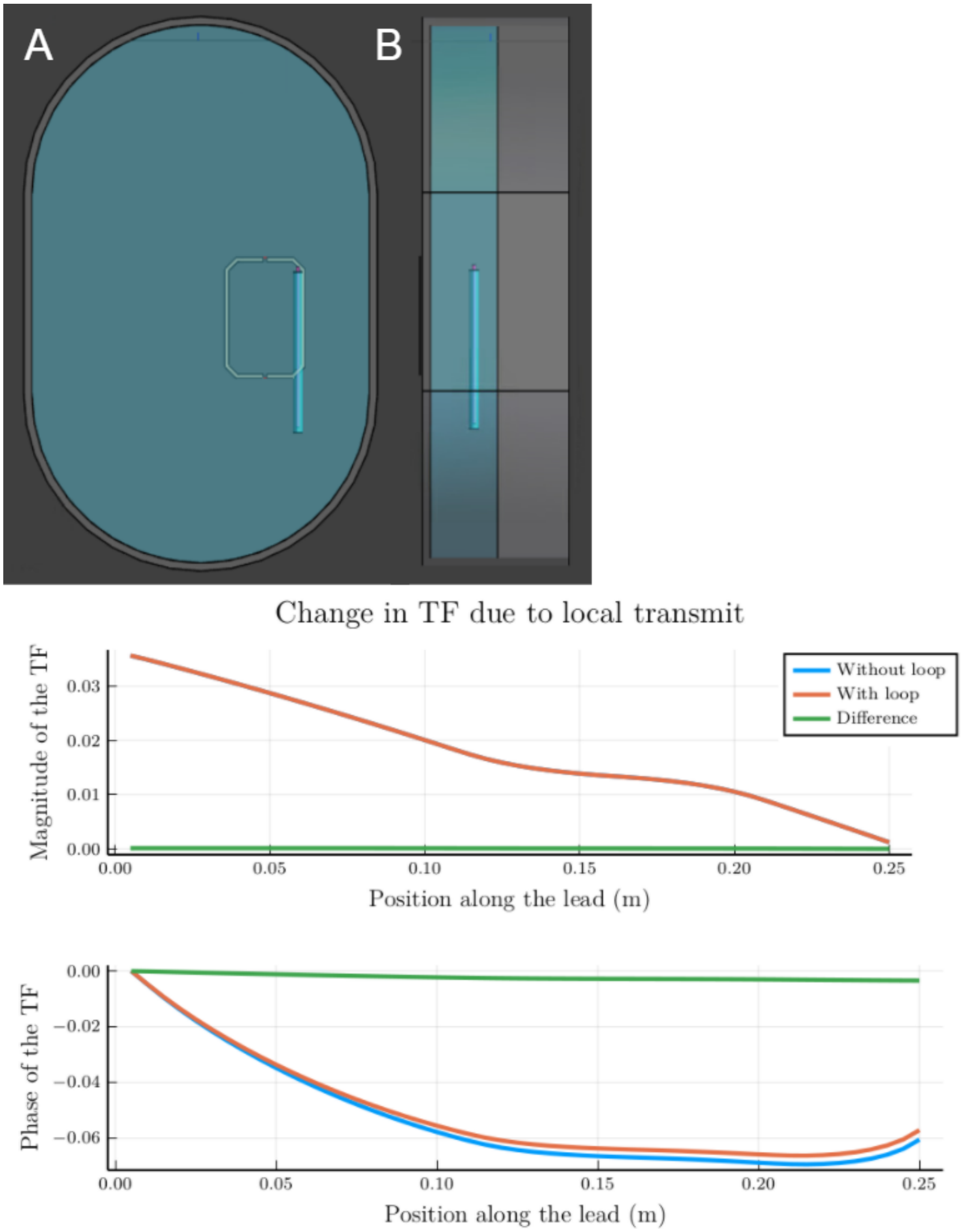

Supporting Information Figure S2: A) shows the top view of the simulation setup and B) shows the side view, where the local transmit coil is placed directly underneath the phantom. The TF was simulated for the setup shown in A) & B) with and without the local transmit coil present. Below the setup the magnitude and the phase of the simulated TFs are shown. Which are in good agreement, only a negligible change in the phase.
